# Supplementary material for: Uncovering the Grinnellian niche space of the cryptic species complex Gammarus roeselii
Source: PeerJ. 2023 Aug 3;11:e15800. doi: 10.7717/peerj.15800 (PMC10404395; doi:10.7717/peerj.15800)
Supplement: Supplemental Information 1 — MOTUs follow the naming of Grabowski et al. (2017). Sampling locations are given in decimal degree. If sampling sites are the same or close (<2 km) to the ones provided in Grabowski et al. (2017) the corresponding ID is supplied. The coordinates were used in the making of the map in Fig. 1. Country abbreviations: AL, Albania; DE, Germany; GR, Greece; SL, Slovenia. [file peerj-11-15800-s001.docx]

| **Site** | **Country** | **Name of the waterbody** | **Type of waterbody** | **MOTU** | **Location North**  **[decimal °]** | **Location East**  **[decimal °]** | **Site ID by Grabowski et al (2017)** |
| --- | --- | --- | --- | --- | --- | --- | --- |
| 1 | DE | Ulmbach | River | C | 50.390583 | 9.400805 | n.a. |
| 2 | DE | Kinzig | River | C | 50.346335 | 9.552046 | n.a. |
| 3 | SL | Drava | River | C | 46.609367 | 14.973586 | n.a. |
| 4 | SL | Drava | River | C | 46.544348 | 15.504712 | n.a. |
| 5 | SL | Mur | River | C | 46.589678 | 16.177896 | n.a. |
| 6 | SL | Drava | River | C | 46.522732 | 16.278691 | n.a. |
| 7 | SL | Scavnica | River | C | 46.522467 | 16.150638 | n.a. |
| 8 | SL | Turja | River | C | 46.533432 | 16.054710 | n.a. |
| 9 | SL | Drava | River | C | 46.379587 | 15.939002 | 2 |
| 10 | AL | Drin | River | G | 42.024833 | 19.519133 | 6 |
| 11 | AL | Shkumbin | River | G | 41.071075 | 20.466527 | 11 |
| 12 | AL | Shkumbin | River | G | 41.064025 | 20.565916 | n.a. |
| 13 | AL | Ohrid | Lake | G | 40.900710 | 20.687130 | n.a. |
| 15 | AL | Ohrid | Lake | G | 41.067533 | 20.644230 | 7 |
| 16 | AL | Devolli | River | G | 40.797563 | 20.718132 | n.a. |
| 17 | AL | Devolli | River | G | 40.657933 | 20.743191 | n.a. |
| 18 | AL | Devolli | River | G | 40.524361 | 20.702765 | n.a. |
| 19 | AL | Devolli | River | G | 40.707898 | 20.871388 | 13 |
| 20 | AL | Prespa | Lake | G | 40.861420 | 20.940791 | 8 |
| 21 | GR | Micri Prespa | Lake | G | 40.745446 | 21.114912 | 10 |
| 22 | GR | Eleshka Reka | River | A | 40.859648 | 21.488888 | n.a. |
| 23 | GR | Papadia | Lake | A | 40.831339 | 21.541287 | n.a. |
| 24 | GR | Papadia | Lake | A | 40.840163 | 21.584415 | n.a. |
| 25 | GR | Petres | Lake | A | 40.728311 | 21.681089 | 17 |
| 26 | GR | Vegoritida | Lake | A | 40.738977 | 21.756498 | 18 |
| 27 | GR | Kastoria | Lake | A | 40.515293 | 21.255458 | 21 |
| 28 | GR | Aliakmonas | River | A | 40.435215 | 21.270994 | n.a. |
| 29 | GR | Aliakmonas | River | A | 40.332211 | 21.411812 | n.a. |
| 30 | GR | Portaikos | River | L | 39.530766 | 21.702878 | n.a. |
| 31 | GR | Portaikos | River | L | 39.542354 | 21.613207 | n.a. |
| 32 | GR | Litheos | River | L | 39.525436 | 21.770519 | n.a. |
| 33 | GR | Litheos | River | L | 39.533338 | 21.925948 | n.a. |
| 34 | GR | Sofaditis | River | K | 39.197062 | 22.032895 | n.a. |
| 35 | GR | Smokovou | Lake | K | 39.120757 | 22.158304 | n.a. |
| 36 | GR | Spercheios | River | K | 38.943423 | 22.211475 | 24 |
| 37 | GR | Spercheios | River | K | 38.938710 | 22.162333 | n.a. |
| 38 | GR | Spercheois | River | K | 38.897608 | 22.317396 | n.a. |
| 39 | GR | Spercheiois | River | K | 38.867853 | 22.363450 | n.a. |
| 40 | GR | Spercheios | River | K | 38.813142 | 22.491720 | n.a. |
| 41 | GR | Smokovou | Lake | K | 39.094976 | 22.194814 | n.a. |
| 42 | GR | Pinios | River | L | 39.878257 | 22.584298 | 25 |
