# Supplementary material for: Uncovering the Grinnellian niche space of the cryptic species complex Gammarus roeselii
Source: PeerJ. 2023 Aug 3;11:e15800. doi: 10.7717/peerj.15800 (PMC10404395; doi:10.7717/peerj.15800)
Supplement: Supplemental Information 2 — Barcode of Life (BOLD) IDs run from CRSEE001-23 to CRSEE0082-23. Primers used are LCO1490 and HCO 2198 (Folmer et al., 1994), UCOIF and UCOIR (Costa et al., 2009) and COIGrF and COIGrR2 (Grabowski et al., 2017) and are further described in the Materials and Methods. [file peerj-11-15800-s002.docx]

| Site ID | ID | BOLD ID | MOTU | Forward Primer | Reverse Primer |
| --- | --- | --- | --- | --- | --- |
| 1 | Cr-01-01 | CRSEE001-23 | C | UCOIF | UCOIR |
| 1 | Cr-01-02 | CRSEE002-23 | C | UCOIF | UCOIR |
| 2 | Cr-02-01 | CRSEE003-23 | C | UCOIF | UCOIR |
| 2 | Cr-02-02 | CRSEE004-23 | C | UCOIF | UCOIR |
| 3 | Cr-03-01 | CRSEE005-23 | C | UCOIF | UCOIR |
| 3 | Cr-03-02 | CRSEE006-23 | C | UCOIF | UCOIR |
| 4 | Cr-04-01 | CRSEE007-23 | C | UCOIF | UCOIR |
| 4 | Cr-04-02 | CRSEE008-23 | C | UCOIF | UCOIR |
| 5 | Cr-05-01 | CRSEE009-23 | C | UCOIF | UCOIR |
| 5 | Cr-05-02 | CRSEE010-23 | C | COIGrF | COIGrR2 |
| 6 | Cr-06-01 | CRSEE011-23 | C | UCOIF | UCOIR |
| 6 | Cr-06-02 | CRSEE012-23 | C | UCOIF | UCOIR |
| 7 | Cr-07-01 | CRSEE013-23 | C | UCOIF | UCOIR |
| 7 | Cr-07-02 | CRSEE014-23 | C | UCOIF | UCOIR |
| 8 | Cr-08-01 | CRSEE015-23 | C | UCOIF | UCOIR |
| 8 | Cr-08-02 | CRSEE016-23 | C | UCOIF | UCOIR |
| 9 | Cr-09-01 | CRSEE017-23 | C | UCOIF | UCOIR |
| 9 | Cr-09-02 | CRSEE018-23 | C | UCOIF | UCOIR |
| 10 | Cr-10-01 | CRSEE019-23 | G | UCOIF | UCOIR |
| 10 | Cr-10-02 | CRSEE020-23 | G | UCOIF | UCOIR |
| 11 | Cr-11-01 | CRSEE021-23 | G | UCOIF | UCOIR |
| 11 | Cr-11-03 | CRSEE022-23 | G | COIGrF | COIGrR2 |
| 12 | Cr-12-01 | CRSEE023-23 | G | UCOIF | UCOIR |
| 12 | Cr-12-02 | CRSEE024-23 | G | UCOIF | UCOIR |
| 13 | Cr-13-01 | CRSEE025-23 | G | UCOIF | UCOIR |
| 13 | Cr-13-02 | CRSEE026-23 | G | UCOIF | UCOIR |
| 15 | Cr-15-01 | CRSEE027-23 | G | UCOIF | UCOIR |
| 15 | Cr-15-02 | CRSEE028-23 | G | UCOIF | UCOIR |
| 16 | Cr-16-01 | CRSEE029-23 | G | UCOIF | UCOIR |
| 16 | Cr-16-02 | CRSEE030-23 | G | UCOIF | UCOIR |
| 17 | Cr-17-01 | CRSEE031-23 | G | UCOIF | UCOIR |
| 17 | Cr-17-02 | CRSEE032-23 | G | UCOIF | UCOIR |
| 18 | Cr-18-01 | CRSEE033-23 | G | UCOIF | UCOIR |
| 18 | Cr-18-02 | CRSEE034-23 | G | UCOIF | UCOIR |
| 19 | Cr-19-01 | CRSEE035-23 | G | UCOIF | UCOIR |
| 19 | Cr-19-02 | CRSEE036-23 | G | UCOIF | UCOIR |
| 20 | Cr-20-01 | CRSEE037-23 | G | UCOIF | UCOIR |
| 20 | Cr-20-02 | CRSEE038-23 | G | UCOIF | UCOIR |
| 21 | Cr-21-01 | CRSEE039-23 | G | UCOIF | UCOIR |
| 21 | Cr-21-02 | CRSEE040-23 | G | UCOIF | UCOIR |
| 22 | Cr-22-01 | CRSEE041-23 | A | UCOIF | UCOIR |
| 22 | Cr-22-02 | CRSEE042-23 | A | UCOIF | UCOIR |
| 23 | Cr-23-01 | CRSEE043-23 | A | UCOIF | UCOIR |
| 23 | Cr-23-02 | CRSEE044-23 | A | UCOIF | UCOIR |
| 24 | Cr-24-01 | CRSEE045-23 | A | UCOIF | UCOIR |
| 24 | Cr-24-02 | CRSEE046-23 | A | UCOIF | UCOIR |
| 25 | Cr-25-01 | CRSEE047-23 | A | UCOIF | UCOIR |
| 25 | Cr-25-02 | CRSEE048-23 | A | UCOIF | UCOIR |
| 26 | Cr-26-01 | CRSEE049-23 | A | UCOIF | UCOIR |
| 26 | Cr-26-02 | CRSEE050-23 | A | UCOIF | UCOIR |
| 27 | Cr-27-01 | CRSEE051-23 | A | UCOIF | UCOIR |
| 27 | Cr-27-02 | CRSEE052-23 | A | UCOIF | UCOIR |
| 28 | Cr-28-01 | CRSEE053-23 | A | UCOIF | UCOIR |
| 28 | Cr-28-02 | CRSEE054-23 | A | UCOIF | UCOIR |
| 29 | Cr-29-01 | CRSEE055-23 | A | UCOIF | UCOIR |
| 29 | Cr-29-02 | CRSEE056-23 | A | UCOIF | UCOIR |
| 30 | Cr-30-01 | CRSEE057-23 | L | UCOIF | UCOIR |
| 30 | Cr-30-02 | CRSEE058-23 | L | UCOIF | UCOIR |
| 31 | Cr-31-01 | CRSEE059-23 | L | UCOIF | UCOIR |
| 31 | Cr-31-02 | CRSEE060-23 | L | UCOIF | UCOIR |
| 32 | Cr-32-01 | CRSEE061-23 | L | UCOIF | UCOIR |
| 32 | Cr-32-05 | CRSEE062-23 | L | COIGrF | COIGrR2 |
| 33 | Cr-33-01 | CRSEE063-23 | L | UCOIF | UCOIR |
| 33 | Cr-33-02 | CRSEE064-23 | L | UCOIF | UCOIR |
| 34 | Cr-34-01 | CRSEE065-23 | K | LCO1490 | HCO2198 |
| 34 | Cr-34-03 | CRSEE066-23 | K | UCOIF | UCOIR |
| 35 | Cr-35-01 | CRSEE067-23 | K | LCO1490 | HCO2198 |
| 35 | Cr-35-02 | CRSEE068-23 | K | LCO1490 | HCO2198 |
| 36 | Cr-36-01 | CRSEE069-23 | K | LCO1490 | HCO2198 |
| 36 | Cr-36-02 | CRSEE070-23 | K | LCO1490 | HCO2198 |
| 37 | Cr-37-01 | CRSEE071-23 | K | LCO1490 | HCO2198 |
| 37 | Cr-37-02 | CRSEE072-23 | K | LCO1490 | HCO2198 |
| 38 | Cr-38-01 | CRSEE073-23 | K | LCO1490 | HCO2198 |
| 38 | Cr-38-02 | CRSEE074-23 | K | LCO1490 | HCO2198 |
| 39 | Cr-39-01 | CRSEE075-23 | K | LCO1490 | HCO2198 |
| 39 | Cr-39-02 | CRSEE076-23 | K | LCO1490 | HCO2198 |
| 40 | Cr-40-01 | CRSEE077-23 | K | LCO1490 | HCO2198 |
| 40 | Cr-40-02 | CRSEE078-23 | K | LCO1490 | HCO2198 |
| 41 | Cr-41-01 | CRSEE079-23 | K | LCO1490 | HCO2198 |
| 41 | Cr-41-02 | CRSEE080-23 | K | LCO1490 | HCO2198 |
| 42 | Cr-42-01 | CRSEE081-23 | L | UCOIF | UCOIR |
| 42 | Cr-42-02 | CRSEE082-23 | L | UCOIF | UCOIR |
