# Supplementary material for: Uncovering the Grinnellian niche space of the cryptic species complex Gammarus roeselii
Source: PeerJ. 2023 Aug 3;11:e15800. doi: 10.7717/peerj.15800 (PMC10404395; doi:10.7717/peerj.15800)
Supplement: Supplemental Information 3 — All G. roeselii sequences were published by Grabowski et al. (2017). The sequence of G. fossarum is published in Wattier et al. (2020) and taken as outgroup. All sequences are available online at the National Center of Biotechnology Information (NCBI). [file peerj-11-15800-s003.docx]

| **Country** | **River/Lake system** | **MOTU** | **NCBI Accession number** | **Voucher** |
| --- | --- | --- | --- | --- |
| AL | Shkoder, Drin River | G | KP789708 | GROE-AL18-1 |
| AL | Shkoder, Drin River | G (GG) | KP789697 | GROE-AL18-4 |
| AL | Shkoder, Drin River | G | KP789709 | GROE-AL18-8 |
| AL | Orikum, Dukatit River | H | KP789720 | GROE-AL33-1 |
| AL | Orikum, Dukatit River | H | KP789723 | GROE-AL33-2 |
| AL | Orikum, Dukatit River | H | KP789721 | GROE-AL33-4 |
| AL | Orikum, Dukatit River | H | KP789722 | GROE-AL33-5 |
| AL | Gollomboc, Prespa Lake | G | KP789704 | GROE-AL34-1 |
| AL | Gollomboc, Prespa Lake | G | KP789705 | GROE-AL34-2 |
| AL | Gollomboc, Prespa Lake | G | KP789698 | GROE-AL34-3 |
| AL | Perrenjas, tributary of Shkumbin | G | KP789707 | GROE-AL35-2 |
| AL | Perrenjas, tributary of Shkumbin | E | KP789691 | GROE-AL35-3 |
| AL | Perrenjas, tributary of Shkumbin | G | KP789700 | GROE-AL35-4 |
| AL | Perrenjas, tributary of Shkumbin | G | KP789701 | GROE-AL35-6 |
| AL | Lin, Ohrid Lake | G | KP789696 | GROE-AL36-6 |
| AL | Petritsio, Strymonas River | E | KP789712 | GROE-AL45-1 |
| AL | Petritsio, Strymonas River | E | KP789714 | GROE-AL45-13 |
| AL | Petritsio, Strymonas River | E | KP789713 | GROE-AL45-15 |
| AL | Zvezde, Devoll River | J | KP789725 | GROE-AL45-17 |
| AL | Zvezde, Devoll River | J | KP789724 | GROE-AL45-20 |
| AL | Petritsio, Strymonas River | E | KP789711 | GROE-AL45-23 |
| RO | Makoviste, Vicinic River | C | KP789693 | GROE-BG01-1 |
| BG | Drangovo-Marikostinovo, Struma | A | KP789683 | GROE-BG29-1 |
| BG | Drangovo-Marikostinovo, Struma | A | KP789684 | GROE-BG29-10 |
| BG | Drangovo-Marikostinovo, Struma | A | KP789686 | GROE-BG29-2 |
| BG | Drangovo-Marikostinovo, Struma | A | KP789681 | GROE-BG29-4 |
| BG | Drangovo-Marikostinovo, Struma | A | KP789682 | GROE-BG29-6 |
| CR | Varazdin, tributary of Drava River | A | KP789692 | GROE-CRVAR-8 |
| NM | Canion Matka, Treska River | D | KP789690 | GROE-GR02-1 |
| NM | Canion Matka, Treska River | A | KP789679 | GROE-GR02-10 |
| NM | Otesevo, Prespa Lake | A | KP789702 | GROE-GR04-1 |
| NM | Otesevo, Prespa Lake | A | KP789699 | GROE-GR04-3 |
| GR | Otesevo, Prespa Lake | A | KP789706 | GROE-GR04-5 |
| GR | Microlimni Micri, Prespa Lake | A | KP789703 | GROE-GR05-6 |
| GR | Kastoria, Kastoria (Orestiada) Lake | A | KP789677 | GROE-GR07-1 |
| GR | Kastoria, Kastoria (Orestiada) Lake | A | KP789678 | GROE-GR07-11 |
| GR | Petres, Petron Lake | A | KP789673 | GROE-GR09-11 |
| GR | Petres, Petron Lake | A | KP789674 | GROE-GR09-13 |
| GR | Petres, Petron Lake | A | KP789680 | GROE-GR09-8 |
| GR | Agios Panteleimon, Vegoritis Lake | A | KP789688 | GROE-GR10-11 |
| GR | Agios Panteleimon, Vegoritis Lake | A | KP789676 | GROE-GR10-2 |
| GR | Agios Panteleimon, Vegoritis Lake | A | KP789689 | GROE-GR10-4 |
| GR | Agios Panteleimon, Vegoritis Lake | A | KP789675 | GROE-GR10-6 |
| GR | Agios Panteleimon, Vegoritis Lake | A | KP789687 | GROE-GR10-9 |
| GR | Soulopoulo, tributary of Kalamas (Thyamis) River | M | KP789744 | GROE-GR14-10 |
| GR | Soulopoulo, tributary of Kalamas (Thyamis) River | M | KP789746 | GROE-GR14-11 |
| GR | Soulopoulo, tributary of Kalamas (Thyamis) River | M | KP789742 | GROE-GR14-12 |
| GR | Soulopoulo, tributary of Kalamas (Thyamis) River | M | KP789740 | GROE-GR14-3 |
| GR | Soulopoulo, tributary of Kalamas (Thyamis) River | M | KP789745 | GROE-GR14-4 |
| GR | Soulopoulo, tributary of Kalamas (Thyamis) River | M | KP789741 | GROE-GR14-5 |
| GR | Soulopoulo, tributary of Kalamas (Thyamis) River | M | KP789743 | GROE-GR14-9 |
| GR | Platanias, Trichonida (Trichonis) | I | KP789726 | GROE-GR19 |
| GR | Platanias, Trichonida (Trichonis) | I | KP789729 | GROE-GR19-1 |
| GR | Platanias, Trichonida (Trichonis) | I | KP789727 | GROE-GR19-10 |
| GR | Platanias, Trichonida (Trichonis) | I | KP789730 | GROE-GR19-2 |
| GR | Platanias, Trichonida (Trichonis) | I | KP789731 | GROE-GR19-3 |
| GR | Platanias, Trichonida (Trichonis) | I | KP789728 | GROE-GR19-8 |
| GR | Kedros, Sofaditikos River | K | KP789735 | GROE-GR24-1 |
| GR | Kedros, Sofaditikos River | K | KP789734 | GROE-GR24-10 |
| GR | Kedros, Sofaditikos River | K | KP789732 | GROE-GR25-1 |
| GR | Kedros, Sofaditikos River | K | KP789733 | GROE-GR25-12 |
| GR | Omolio, Pinios River | L | KP789736 | GROE-GR30-1 |
| GR | Omolio, Pinios River | L | KP789739 | GROE-GR30-12 |
| GR | Omolio, Pinios River | L | KP789737 | GROE-GR30-2 |
| GR | Omolio, Pinios River | L | KP789738 | GROE-GR30-3 |
| GR | Aleksandria, Aliakmonas River | F | KP789719 | GROE-GR33-1 |
| GR | Aleksandria, Aliakmonas River | F | KP789718 | GROE-GR33-11 |
| GR | Aleksandria, Aliakmonas River | F | KP789717 | GROE-GR33-12 |
| GR | Aleksandria, Aliakmonas River | F | KP789716 | GROE-GR33-4 |
| GR | Petritsio, Strymonas River | E | KP789710 | GROE-GR38-10 |
| GR | Petritsio, Struma (Strymonas) River | A | KP789685 | GROE-GR38-2 |
| SL | Creta, tributary of Drava River | C | KP789695 | GROE-SLODRA-11 |
| SL | Nova Vas, Drava River | C | KP789694 | GROE-SLONVD-4 |
| SR | Ilince near Presevo, tributary of Binacka Morava River | B | KP789715 | GROE-SRBPRE-6 |
| SL | Ljubljanica, Danube | *Gammarus fossarum* | MT978847 | NITAM00417 |
