# Supplementary material for: Uncovering the Grinnellian niche space of the cryptic species complex Gammarus roeselii
Source: PeerJ. 2023 Aug 3;11:e15800. doi: 10.7717/peerj.15800 (PMC10404395; doi:10.7717/peerj.15800)
Supplement: Supplemental Information 4 — The between each MOTU K2P distance is situated in the bottom left and standard error in the upper right corner of the matrix. K2P distance is calculated after Kimura (1980). [file peerj-11-15800-s004.docx]

|  | **MOTU C** | **MOTU G** | **MOTU A** | **MOTU L** | **MOTU K** |
| --- | --- | --- | --- | --- | --- |
| **MOTU C** |  | 0.0146 | 0.0110 | 0.0229 | 0.0234 |
| **MOTU G** | 0.1068 |  | 0.0156 | 0.0208 | 0.0219 |
| **MOTU A** | 0.0799 | 0.1260 |  | 0.0220 | 0.0232 |
| **MOTU L** | 0.2279 | 0.1860 | 0.2165 |  | 0.0160 |
| **MOTU K** | 0.2294 | 0.1931 | 0.2269 | 0.1160 |  |
