# Supplementary material for: Uncovering the Grinnellian niche space of the cryptic species complex Gammarus roeselii
Source: PeerJ. 2023 Aug 3;11:e15800. doi: 10.7717/peerj.15800 (PMC10404395; doi:10.7717/peerj.15800)
Supplement: Supplemental Information 5 — K2P distance is calculated within each MOTU and the standard error is given below each value. K2P distance is calculated after Kimura (1980). [file peerj-11-15800-s005.docx]

|  | **MOTU C** | **MOTU G** | **MOTU A** | **MOTU L** | **MOTU K** |
| --- | --- | --- | --- | --- | --- |
| **within group mean distance** | 0.0219 | 0.0097 | 0.0206 | 0.0160 | 0.0020 |
| **S.E.** | 0.0044 | 0.0029 | 0.0041 | 0.0035 | 0.0012 |
