# Supplementary material for: Uncovering the Grinnellian niche space of the cryptic species complex Gammarus roeselii
Source: PeerJ. 2023 Aug 3;11:e15800. doi: 10.7717/peerj.15800 (PMC10404395; doi:10.7717/peerj.15800)
Supplement: Supplemental Information 6 — Raw data of each environmental and chemical variable and their values are listed here. The corresponding units are displayed in brackets. All data displayed was used to evaluate the PCA and the niches for each MOTU. If the value of the estrogen or dioxin-like activity was below the limit of quantification (LOQ) it is stated as <LOQ. LOQ for the YES = 0.305 ng/g; LOQ for YDS = 0.092 mg/g. In the analysis it was set to zero to account for no estrogenic or dioxin-like activity. [file peerj-11-15800-s006.docx]

| **Site** | **Altitude [m]** | | **Mean flow velocity [m/s]** | **pH** | **Conductivity [µS/cm]** | **O_2_ saturation [%]** | **Temperature [°C]** | **Nitrate [mg/L]** | **Nitrite [mg/L]** | **Phosphate [mg/L]** | **Carbonate hardness [°dH]** | **YES [ng/g]** | **YDS [mg/g]** | **EC_50_-100  [mg sediment equivalency]** |
| --- | --- | --- | --- | --- | --- | --- | --- | --- | --- | --- | --- | --- | --- | --- |
| 1 | 400 | | 0.05 | 9.76 | 225.0 | 9.75 | 14 | 28 | 0.00 | 0.24 | 1.6 | < LOQ | 1.105 | 88.68 |
| 2 | 223 | | 0.26 | 8.19 | 510.0 | 10.53 | 14 | 12 | 0.00 | 0.12 | 14.4 | < LOQ | 4.070 | 65.73 |
| 3 | 343 | | 0.01 | 7.53 | 253.0 | 9.23 | 15.7 | 0 | 0.00 | 0.00 | 6.0 | < LOQ | 0.165 | 7.38 |
| 4 | 247 | | 0.02 | 7.63 | 255.0 | 9.32 | 17.3 | 0 | 0.00 | 0.00 | 6.2 | 0.654 | 4.600 | 95.31 |
| 5 | 184 | | 0.35 | 7.80 | 313.0 | 10.53 | 15.3 | 0 | 0.00 | 0.02 | 6.6 | 0.629 | 2.130 | 83.40 |
| 6 | 170 | | 0.03 | 7.77 | 441.0 | 10.33 | 20.2 | 0 | 0.00 | 0.00 | 11.0 | 1.102 | 0.285 | 96.17 |
| 7 | 178 | | 0.32 | 7.88 | 414.0 | 10.11 | 21.9 | 1 | 0.05 | 0.00 | 15.4 | 1.221 | 0.194 | 97.78 |
| 8 | 204 | | 0.20 | 7.87 | 544.0 | 8.77 | 16.5 | 4 | 0.05 | 0.50 | 11.4 | < LOQ | 0.271 | 69.07 |
| 9 | 211 | | 0.34 | 7.95 | 270.0 | 10.17 | 17 | 2 | 0.02 | 0.14 | 6.4 | 7.916 | 3.320 | 96.70 |
| 10 | 12 | | 0.37 | 7.74 | 278.0 | 8.56 | 24.4 | 0 | 0.00 | 0.27 | 7.2 | < LOQ | 0.486 | 87.28 |
| 11 | 411 | | 0.20 | 8.54 | 565.0 | 11.37 | 20.8 | 0 | 0.01 | 0.19 | 18.2 | < LOQ | 0.209 | 72.17 |
| 12 | 578 | | 0.46 | 8.01 | 486.0 | 8.39 | 20.4 | 5 | 0.06 | 0.32 | 16.2 | < LOQ | 0.440 | 49.27 |
| 13 | 692 | | 0.00 | 8.21 | 216.2 | 10.56 | 23.4 | 0 | 0.02 | 0.05 | 5.4 | 3.229 | 6.970 | 96.60 |
| 15 | 696 | | 0.09 | 8.17 | 222.0 | 9.85 | 22.8 | 0 | 0.01 | 0.22 | 6.2 | < LOQ | 0.800 | 94.57 |
| 16 | 832 | | 0.13 | 8.08 | 500.0 | 10.31 | 17.1 | 11 | 0.25 | 0.40 | 14.2 | 2.862 | 2.010 | 97.71 |
| 17 | 825 | | 0.15 | 8.27 | 430.0 | 7.48 | 19 | 3 | 0.03 | 0.18 | 14.0 | 0.318 | 1.070 | 0.00 |
| 18 | 905 | | 0.31 | 8.25 | 473.0 | 8.68 | 17.4 | 0 | 0.01 | 0.50 | 15.6 | 0.392 | 0.445 | 95.44 |
| 19 | 826 | | 0.32 | 8.19 | 563.0 | 10.79 | 19.7 | 0 | 0.04 | 0.15 | 18.2 | < LOQ | < LOQ | 96.24 |
| 20 | 847 | | 0.00 | 8.60 | 210.6 | 12.53 | 24 | 0 | 0.00 | 0.00 | 5.6 | 0.769 | 0.330 | 95.85 |
| 21 | 851 | | 0.00 | 8.42 | 254.0 | 11.6 | 19.3 | 0 | 0.00 | 0.10 | 7.4 | < LOQ | 1.690 | 53.68 |
| 22 | 596 | | 0.03 | 8.02 | 374.0 | 5.76 | 16.2 | 20 | 0.94 | 0.78 | 5.4 | < LOQ | 0.330 | 94.90 |
| 23 | 628 | | 0.00 | 8.07 | 377.0 | 8.42 | 19.6 | 0 | 0.06 | 0.16 | 7.6 | 5.628 | 2.830 | 96.35 |
| 24 | 668 | | 0.00 | 8.39 | 342.0 | 9.32 | 17.1 | 0 | 0.02 | 0.08 | 5.6 | 1.076 | 0.466 | 74.57 |
| 25 | 574 | | 0.00 | 8.67 | 1100.0 | 14.78 | 21.4 | 0 | 0.00 | 0.01 | 19.0 | < LOQ | 0.171 | 89.33 |
| 26 | 517 | | 0.00 | 8.85 | 639.0 | 13.02 | 22.1 | 0 | 0.01 | 0.03 | 11.0 | 0.427 | < LOQ | 72.58 |
| 27 | 628 | | 0.00 | 8.66 | 292.0 | 8.58 | 21.3 | 0 | 0.02 | 0.00 | 4.8 | 4.819 | 17.900 | 97.49 |
| 28 | 613 | | 0.35 | 8.19 | 452.0 | 8.87 | 16 | 12 | 0.12 | 0.21 | 12.6 | 1.220 | 0.686 | 98.61 |
| 29 | 554 | | 0.49 | 8.30 | 495.0 | 10.87 | 19.1 | 9 | 0.03 | 0.32 | 12.0 | 0.457 | 0.143 | 77.00 |
| 30 | 114 | | 0.13 | 7.91 | 489.0 | 7.32 | 19.8 | 5 | 0.05 | 0.20 | 14.4 | 0.342 | 0.238 | 91.65 |
| 31 | 129 | | 0.22 | 8.17 | 368.0 | 10.2 | 18.7 | 0 | 0.00 | 0.18 | 11.0 | 0.395 | 0.546 | 0.00 |
| 32 | 100 | | 0.04 | 8.10 | 578.0 | 8.94 | 22.6 | 18 | 0.06 | 0.15 | 16.2 | < LOQ | 0.187 | 58.53 |
| 33 | 92 | | 0.25 | 7.96 | 590.0 | 6.01 | 22.3 | 20 | 0.14 | 1.61 | 15.6 | 2.194 | < LOQ | 96.77 |
| 34 | 183 | | 0.26 | 8.49 | 461.0 | 12.4 | 14.9 | 0 | 0.03 | 0.05 | 12.6 | < LOQ | 0.652 | 35.44 |
| 35 | 392 | | 0.00 | 9.01 | 697.0 | 14.23 | 21.4 | 20 | 0.51 | 0.17 | 21.6 | < LOQ | 0.865 | 98.75 |
| 36 | 86 | | 0.05 | 8.00 | 635.0 | 12.46 | 23.1 | 10 | 0.04 | 0.09 | 16.2 | < LOQ | 0.421 | 96.42 |
| 37 | 100 | | 0.12 | 7.85 | 497.0 | 9.98 | 22.3 | 0 | 0.02 | 0.02 | 13.2 | < LOQ | 0.222 | 75.00 |
| 38 | 33 | | 0.23 | 8.02 | 564.0 | 9.19 | 18.5 | 5 | 0.01 | 0.04 | 14.6 | < LOQ | < LOQ | 97.08 |
| 39 | 23 | | 0.43 | 8.09 | 561.0 | 9.53 | 18.1 | 5 | 0.00 | 0.06 | 15.0 | 0.430 | 0.864 | 79.00 |
| 40 | 7 | | 0.33 | 8.09 | 467.0 | 8.51 | 19.7 | 2 | 0.05 | 0.11 | 13.8 | < LOQ | 0.226 | 78.50 |
| 41 | 463 | | 0.00 | 8.27 | 823.0 | 13.6 | 19.8 | 100 | 1.64 | 0.13 | 25.4 | < LOQ | 0.338 | 64.20 |
| 42 | 13 | | 0.02 | 7.73 | 729.0 | 9.4 | 19.1 | 0 | 0.01 | 0.50 | 18.2 | < LOQ | 0.265 | 41.93 |
|  | |  |  |  |  |  |  |  |  |  |  |  |  |  |
| **Min.** | 7 | | 0 | 7.53 | 210.6 | 5.76 | 14 | 0 | 0 | 0 | 1.6 | 0.318 | 0.143 | 0 |
| **Max.** | 905 | | 0.49 | 9.76 | 1100 | 14.78 | 24.4 | 100 | 1.64 | 1.61 | 25.4 | 7.916 | 17.9 | 98.75 |
| **Mean** | 393.85 | | 0.16 | 8.19 | 462.26 | 10.01 | 19.35 | 7.12 | 0.11 | 0.20 | 12.02 | 1.80 | 1.57 | 76.91 |
| **SD** | 284.98 | | 0.15 | 0.40 | 180.87 | 1.95 | 2.75 | 16.34 | 0.29 | 0.28 | 5.24 | 2.05 | 3.09 | 26.74 |
