# Supplementary material for: Uncovering the Grinnellian niche space of the cryptic species complex Gammarus roeselii
Source: PeerJ. 2023 Aug 3;11:e15800. doi: 10.7717/peerj.15800 (PMC10404395; doi:10.7717/peerj.15800)
Supplement: Supplemental Information 7 — Raw data of climatic variables and values are listed here. The corresponding units are displayed in brackets. This raw data was attained from Domisch, Amatulli & Jetz (2015). [file peerj-11-15800-s007.docx]

| **Site** | **Flow length [count of grid cells]** | **Cultivated land cover "lc_avg_07",  [% cover]** | **Urban land cover "lc_avg_09",  [% cover]** | **Annual mean temperature "Bioclim1", [°C]** | **Annual precipitation "Bioclim12", [mm]** |
| --- | --- | --- | --- | --- | --- |
| 1 | 20.00 | 65.00 | 0.00 | 8.25 | 1509.00 |
| 2 | 125.00 | 52.00 | 0.00 | 8.60 | 1412.00 |
| 3 | 20494.00 | 19.00 | 1.00 | 6.40 | 2334.00 |
| 4 | 22637.00 | 14.00 | 1.00 | 6.50 | 2316.00 |
| 5 | 17844.00 | 31.00 | 2.00 | 6.50 | 2068.00 |
| 6 | 18456.00 | 37.00 | 1.00 | 6.60 | 2016.00 |
| 7 | 368.00 | 64.00 | 0.00 | 9.80 | 1784.00 |
| 8 | 263.00 | 58.00 | 0.00 | 9.70 | 1802.00 |
| 9 | 24730.00 | 31.00 | 2.00 | 6.80 | 2208.00 |
| 10 | 20445.00 | 62.00 | 1.00 | 10.80 | 2414.00 |
| 11 | 802.00 | 73.00 | 1.00 | 11.20 | 1940.00 |
| 12 | 49.00 | 73.00 | 5.00 | 11.90 | 1858.00 |
| 13 | 358.00 | 29.00 | 1.00 | 11.10 | 1730.00 |
| 15 | 358.00 | 29.00 | 1.00 | 11.10 | 1730.00 |
| 16 | 65.00 | 91.00 | 3.00 | 10.40 | 1772.00 |
| 17 | 460.00 | 72.00 | 6.00 | 10.30 | 1760.00 |
| 18 | 27.00 | 56.00 | 0.00 | 10.10 | 1818.00 |
| 19 | 2722.00 | 63.00 | 1.00 | 9.90 | 1638.00 |
| 20 | 51.00 | 83.00 | 0.00 | 10.10 | 1694.00 |
| 21 | 1383.00 | 41.00 | 0.00 | 9.90 | 1558.00 |
| 22 | 1023.00 | 67.00 | 1.00 | 10.50 | 1272.00 |
| 23 | 315.00 | 61.00 | 1.00 | 9.90 | 1246.00 |
| 24 | 225.00 | 60.00 | 2.00 | 9.40 | 1278.00 |
| 25 | 365.00 | 53.00 | 2.00 | 11.30 | 1164.00 |
| 26 | 1818.00 | 56.00 | 2.00 | 11.20 | 1162.00 |
| 27 | 248.00 | 48.00 | 2.00 | 11.00 | 1392.00 |
| 28 | 1851.00 | 66.00 | 2.00 | 10.20 | 1552.00 |
| 29 | 3111.00 | 65.00 | 1.00 | 10.60 | 1480.00 |
| 30 | 459.00 | 70.00 | 4.00 | 14.90 | 1570.00 |
| 31 | 56.00 | 63.00 | 0.00 | 15.60 | 1602.00 |
| 32 | 449.00 | 64.00 | 13.00 | 15.20 | 1408.00 |
| 33 | 3310.00 | 64.00 | 3.00 | 14.30 | 1504.00 |
| 34 | 770.00 | 33.00 | 0.00 | 14.70 | 1518.00 |
| 35 | 332.00 | 45.00 | 1.00 | 14.70 | 1460.00 |
| 36 | 835.00 | 38.00 | 0.00 | 15.50 | 1536.00 |
| 37 | 714.00 | 48.00 | 1.00 | 15.30 | 1572.00 |
| 38 | 1578.00 | 43.00 | 1.00 | 15.40 | 1550.00 |
| 39 | 1759.00 | 42.00 | 1.00 | 15.60 | 1530.00 |
| 40 | 2148.00 | 42.00 | 2.00 | 15.70 | 1504.00 |
| 41 | 303.00 | 56.00 | 1.00 | 14.70 | 1456.00 |
| 42 | 16093.00 | 60.00 | 2.00 | 15.00 | 1168.00 |
| Min. | 20 | 14 | 0 | 6.4 | 1162 |
| Max. | 24730 | 91 | 13 | 15.7 | 2414 |
| Mean | 4132.17 | 53.34 | 1.66 | 11.38 | 1641.10 |
| SD | 7378.27 | 16.81 | 2.23 | 2.89 | 309.84 |
