# Supplementary material for: Uncovering the Grinnellian niche space of the cryptic species complex Gammarus roeselii
Source: PeerJ. 2023 Aug 3;11:e15800. doi: 10.7717/peerj.15800 (PMC10404395; doi:10.7717/peerj.15800)
Supplement: Supplemental Information 8 — The loadings of the variables determine the strength of influence on the data distribution. The cumulative explained variance (%) is given below. [file peerj-11-15800-s008.docx]

|  | **PC1** | **PC2** | **PC3** | **PC4** | **PC5** | **PC6** |
| --- | --- | --- | --- | --- | --- | --- |
| **Altitude** | **-0.003** | **0.675** | **0.257** | **0.046** | **0.427** | **-0.082** |
| **Mean flow velocity** | **0.098** | **-0.530** | **0.199** | **0.155** | **0.176** | **0.482** |
| **pH** | **-0.335** | **0.704** | **-0.019** | **0.244** | **0.111** | **0.057** |
| **Conductivity** | **-0.781** | **-0.184** | **-0.217** | **0.003** | **-0.192** | **0.170** |
| **O_2_ saturation** | **-0.311** | **0.367** | **-0.748** | **0.111** | **0.065** | **0.155** |
| **Nitrate, NO_3-_** | **-0.584** | **0.082** | **-0.088** | **-0.678** | **0.135** | **-0.149** |
| **Nitrite, NO_2-_** | **-0.534** | **0.110** | **-0.063** | **-0.739** | **0.167** | **-0.142** |
| **Phosphate** | **-0.215** | **-0.281** | **0.652** | **-0.282** | **0.107** | **0.162** |
| **Carbonate hardness, HCO_3-_** | **-0.771** | **-0.341** | **-0.174** | **-0.037** | **-0.074** | **0.158** |
| **Estrogenic activity** | **0.434** | **0.310** | **0.285** | **-0.323** | **-0.421** | **0.168** |
| **Dioxin-like acitvity** | **0.379** | **0.500** | **0.116** | **-0.165** | **-0.518** | **-0.065** |
| **Flow length** | **0.639** | **-0.395** | **-0.310** | **-0.335** | **-0.001** | **-0.059** |
| **Annual mean precipitation** | **0.666** | **-0.360** | **-0.179** | **-0.201** | **0.323** | **0.004** |
| **Cultivated land cover** | **-0.463** | **0.072** | **0.611** | **0.155** | **0.294** | **0.045** |
| **Urban land cover** | **-0.185** | **-0.244** | **0.366** | **0.070** | **-0.289** | **-0.527** |
| **Annual mean temp** | **-0.678** | **-0.261** | **0.024** | **0.172** | **-0.424** | **0.001** |
| **Baseline toxicity** | **0.082** | **0.291** | **0.125** | **-0.296** | **-0.182** | **0.686** |
|  | | | | | | |
| **Cumulative explained variance [%]** | **24.093** | **38.947** | **50.558** | **60.298** | **67.908** | **74.998** |
