# Supplementary figures and images for: Uncovering the Grinnellian niche space of the cryptic species complex Gammarus roeselii
Source: PeerJ. 2023 Aug 3;11:e15800. doi: 10.7717/peerj.15800 (PMC10404395; doi:10.7717/peerj.15800)

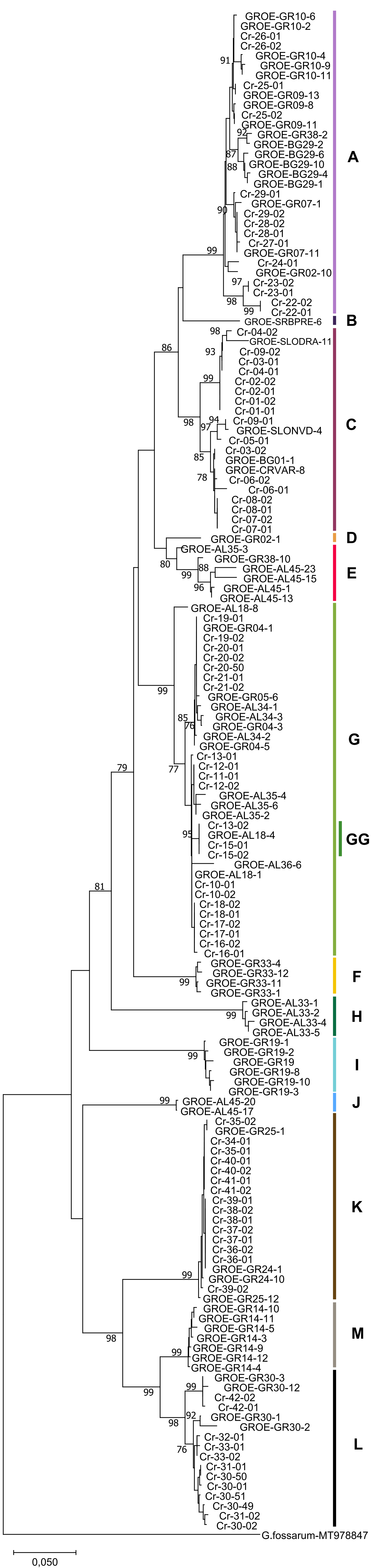

Supplement: Supplemental Information 9 — The neighbour-joining tree was calculated using the bootstrap method with 1,000 iterations with the percentage (>70) of replicate trees on each node. MOTU delimitation follows the sequences determined by Grabowski et al. (2017). The evolutionary distances were computed using the Kimura 2-parameter method with the units of the number of base substitions per site. Newly acquired sequences are abbreviated with “Cr-Site number-Individual Number”. The corresponding site numbers and additional sequences used are listed in in the Supplements. G. fossarum is taken as outgroup. [file peerj-11-15800-s009.pdf]

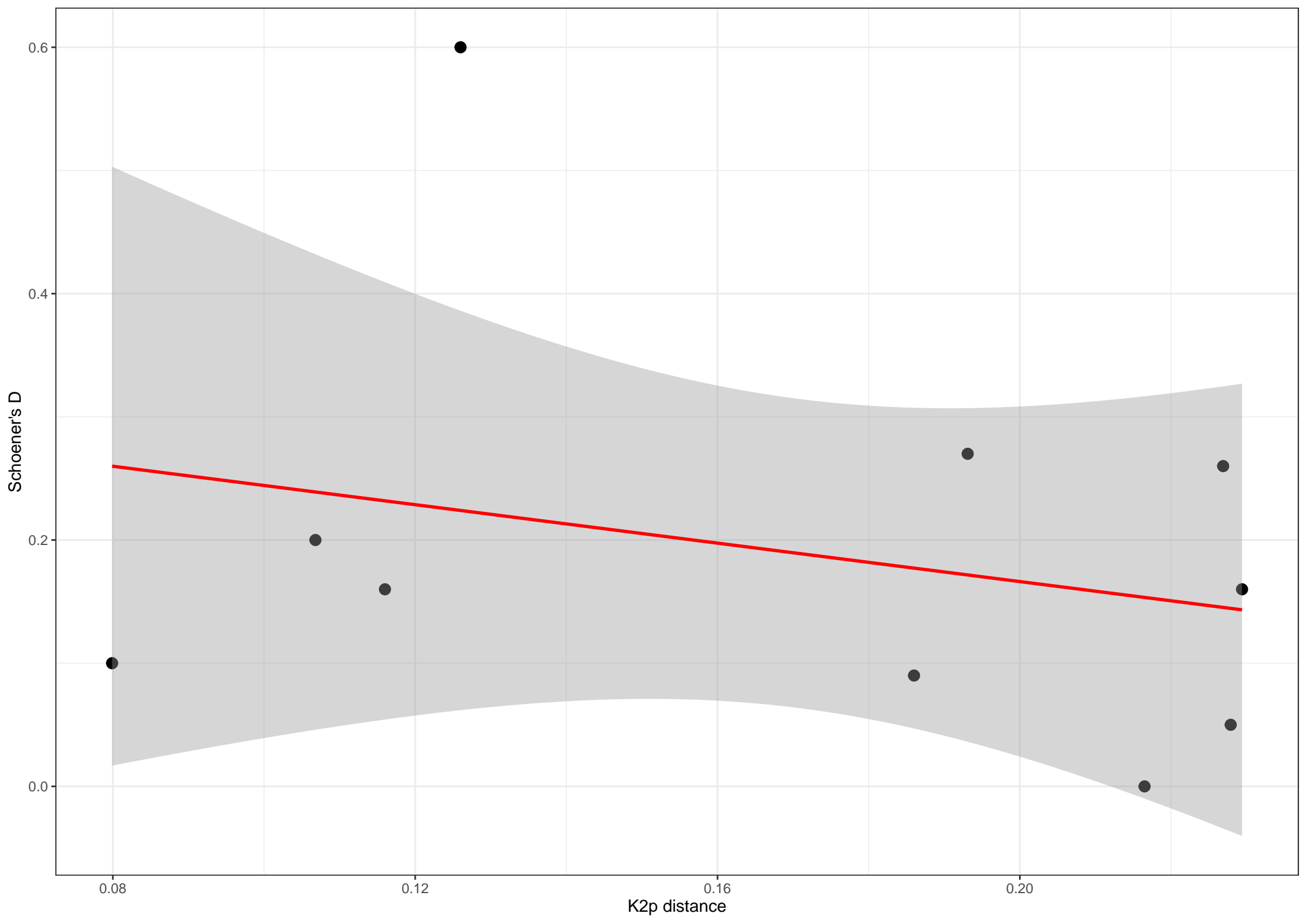

Supplement: Supplemental Information 10 — K2p distance was calculated in MEGA X and represents the genetic distance between each MOTU. Schoener’s D is taken as a measure for niche overlap. Pearson’s r is 0.2677 and p-value is not significant (p-value = 0.4546) marking the linear regression of the two variables as not significant. [file peerj-11-15800-s010.pdf]

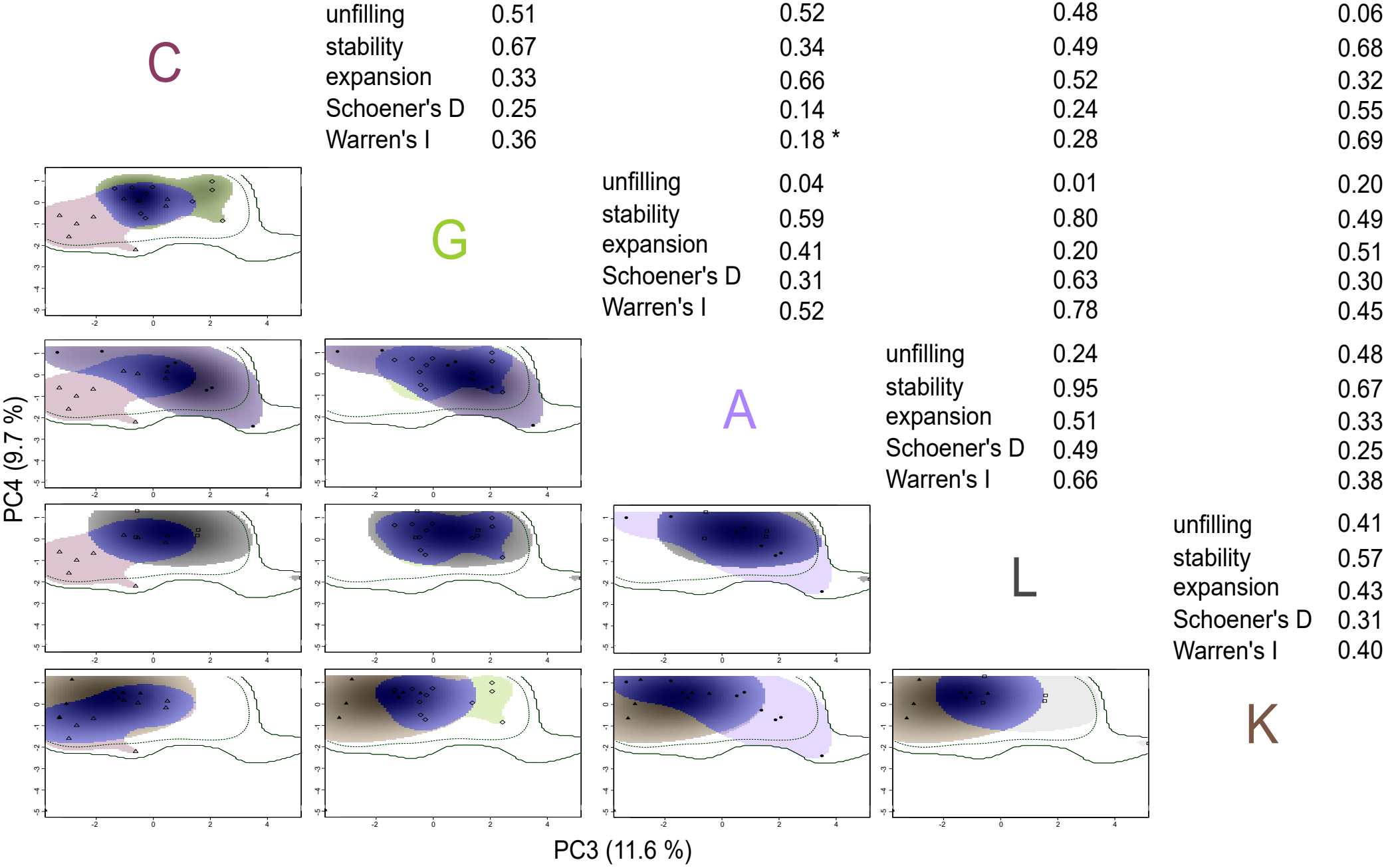

Supplement: Supplemental Information 12 — The niches are spanned along the PC3 and PC4 analysed by the PCA. The outline in each niche plot represents the sensu lato niche space of all analysed data points whereas the dotted inner line is the area in which 75% of the datapoints are situated. The colour of the niches correspond to the according MOTU. Blue coloration is the proportion of overlap where both MOTUs are present. Each marker within the corresponding MOTU represents a sampling site with all its collected data. Density of the occurrence of the second MOTU is illustrated by the shaded area. Calculations are always correspondent to the first MOTU taken into account as explained in the Material and Methods section (“PCA and Niche-Modelling”). Asterisks represent the significance level (*p < 0.05; **p < 0.01; no asterisk = not significant). For all MOTUs combined: mean I = 0.4694 and mean D = 0.3471). [file peerj-11-15800-s012.pdf]
