# Supplementary material for: Uncovering the Grinnellian niche space of the cryptic species complex Gammarus roeselii
Source: PeerJ. 2023 Aug 3;11:e15800. doi: 10.7717/peerj.15800 (PMC10404395; doi:10.7717/peerj.15800)
Supplement: Supplemental Information 11 — Standardized PC3 explains 11.6% and standardized PC4 explains 9.7% of variance. The light grey area represents the Grinnellian niche space of G. roeselii regular lato of our acquired variables. Dark grey is the area in which 75% of the data is gathered. Black dots represent the data for each sampling site. The lengths of the arrows indicate loadings on the principal components. Blue arrows = climatic and geographical variables, red arrows = pollution variables. [file peerj-11-15800-s011.pdf]

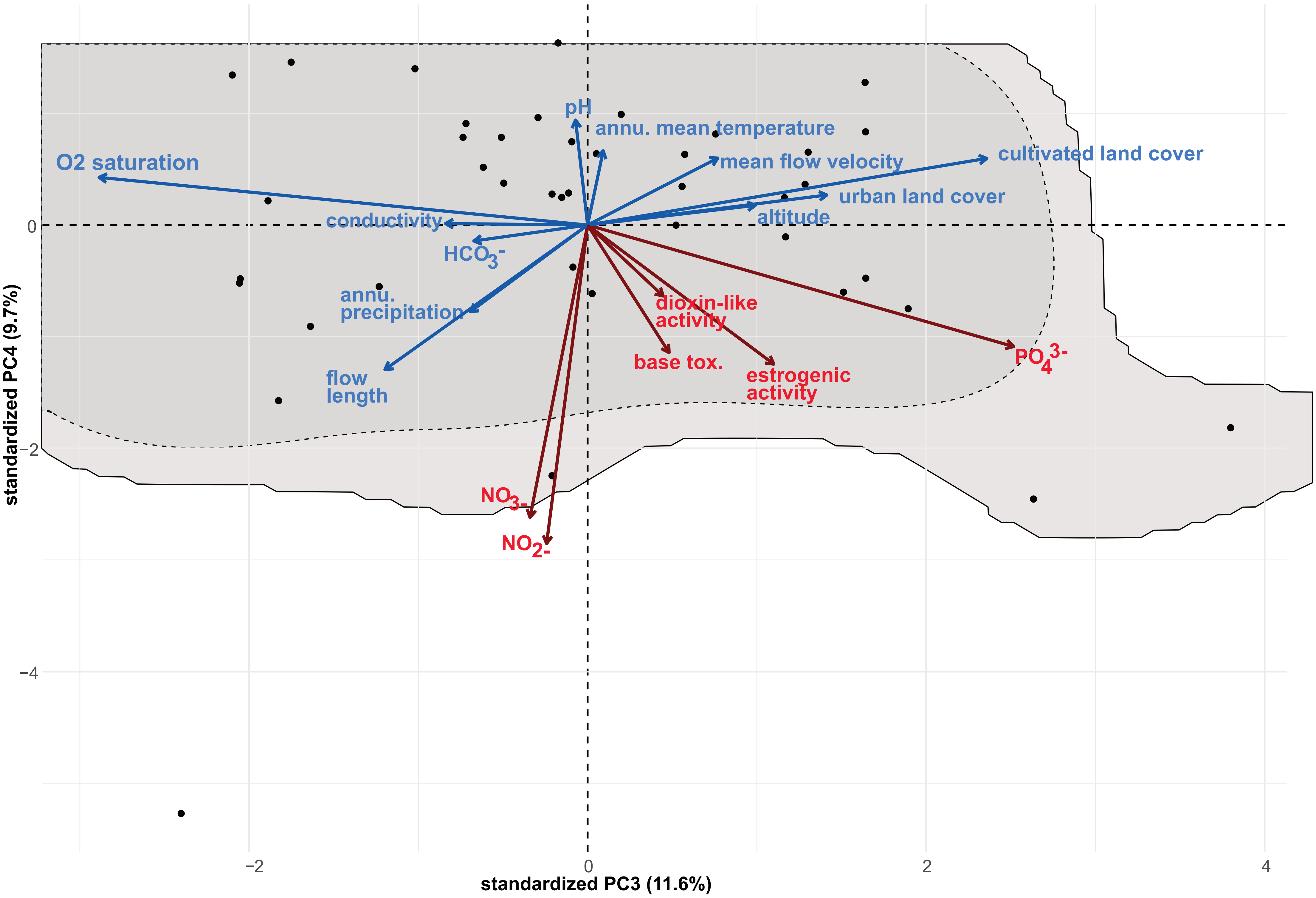

O2 saturation
pH
annu. mean temperature
mean flow velocity
cultivated land cover
urban land cover
altitude
conductivity
HCO_3^-
annu. precipitation
flow length
dioxin-like activity
base tox.
estrogenic activity
PO_4^{3-}
NO_{3-}
NO_{2-}
standardized PC4 (9.7%)
standardized PC3 (11.6%)
0
−2
−4
2
4
